# Charges of Medical Breakdown: The Official Reply

**Published:** 1919-08-23

**Authors:** 


### CHOLERA IN AFGHAN WAR.

### Charges of Medical Breakdown: The Official Reply.

The following is the *Times* summary of the official reply to the disclosures in the *Times* and in Parliament about the medical breakdown in the Afghan Frontier Force as contained in a White Paper [Cmd. 310] issued on the 16th inst. :—

One of the most serious features of the situation was the outbreak of cholera, in regard to which we stated at the end of July that there were 1,663 cases. A dispatch from the Viceroy confirms the statement made that the British troops were but little affected, and gives the actual number of cases down to August 5—namely, 95 British and 1,374 Indian; or 0.59 and 0.88 per cent. of the total numbers employed.

The allegations in the leading article in the *Times* were considered so serious that a summary of them was telegraphed to India next day with instructions to report upon them by telegraph. It had been stated that the violent cholera epidemic in June was apparently due to defective arrangements of water supply. The Government of India, in their reply, say :

Taking the allegations in order, there is no indication as to place at which arrangements for water supply are alleged to have been defective, so we will deal with cholera outbreak as a whole.

Firstly, the conditions should be realised—we have six separate lines of operations covering a front of approximately 500 miles long, the local water supply is often by means of open irrigation channels, but piped-water supply for troops is installed in recognised camps. Cholera is endemic amongst civil population in whole area, and there has been the presence of large numbers recently enlisted transport and other followers without any water discipline or ideas of sanitation, together with insufficiency of super-

**Cholera in Afghan War—(*continued*).**

vision for above owing to shortage of experienced British officers and *personnel.*

Arrangements for immediate supply of pure water in such a terrain are impossible to supervise in the case of troops compelled to march during conditions of abnormal heat, and it is to be remarked that incidence of disease was very small amongst British troops.

The number of cholera cases reported to date total 95 British and 1,374 Indian, mostly followers, that is to say, 0.59 per cent. and 0.88 per cent. respectively of total numbers employed.

Protective measures as follows were initiated before outbreak occurred. Supplementary piped-water supply commenced, but work hampered by shortage of pumps, and pipes demanded from England, Mesopotamia, and elsewhere. Water for troops chlorinated.

News of outbreak was purposely not published in Press for military reasons, and we approve of this action. It would have encouraged Afghan *moral* if news had reached them that we were hampered by disease, and it would have discouraged recruitment in India.

The statement that there were only two medical officers available [*i.e.*, at Peshawar] to attend the wounded is without foundation. Twelve were immediately available, whilst many more were close at hand with mobilised units. Between May 15 and May 17, which included the dates between which the most severe fighting took place, approximately, 322 of all ranks of British and Indian were wounded on the Khyber line. The Director of Medical Services, Khyber Front, reported to headquarters that the majority of the British wounded were in Station Hospital, Peshawar, within few hours of the action, and some actually transferred by ambulance train to Rawal Pindi within the course of the day.

Electric light installation with well electrically lit operating theatre is provided at the British General Hospital at Peshawar.

At no time has there been a shortage of rations. At the outbreak of hostilities the authorised reserves of sixty days for the Field Army stock complete, and indeed in excess in regard to some items. The full original scale of Indian field rations was issued at the outset, but was supplemented by additional items which had been included in the light of experience gained in Mesopotamia. Such of these extras as were actually included in the Mesopotamia scale were available in Karachi and Bombay. Overseas Base Reserves were despatched to the frontier within forty-eight hours of mobilisation on being ordered in accordance with our pre-arranged plans. This was followed by immediate purchase of articles necessary to complete rations to the present scale.

SPECIAL DIFFICULTIES.

The Indian Government proceeds to give a broad outline of the difficulties with which it was confronted:

The Afghan outrage came upon us at a time when not only had we reduced ourselves to the narrowest margin of safety in respect of *personnel* and munitions of war, of which India had been stripped to support forces in Mesopotamia, Egypt, Persia, and France, but when we had still further to reduce our strength in demand to the popular demand for demobilisation in the interest of industrial reconstruction both at home and in India.

Moreover, it occurred at a time when our military arrangements had been to a certain extent dislocated by special measures necessary to preserve order in the Punjab and elsewhere, and to deal with a situation which at one time threatened to spread over India. The same facts had affected our railway services, for considerable damage had been done to the railways themselves, and the whole railway administration of Northern India had been in a certain measure dislocated.

The suddenness with which the Afghan situation developed into a crisis and was at once followed by defection of our Frontier Militia, together with the threat of a rebellion in Peshawar itself, deprived us of the measure of security on which we reasonably counted to cover our concentration, since not only had the advanced echelons of our Field Army to be diverted to replace Militia, but they had actually to preserve order in the districts. Consequently, instead of being able to proceed on a deliberate system of rapid concentration, we were forced at once to send troops to every part of the frontier, and so reverse our pre-arranged plan of concentration, which provided that the dispatch of stores and equipment should precede that of troops.

As regards hospitals, you, no doubt, realise that in a country like the North-West Frontier of India, where there are no public buildings that are capable of immediate occupation, there is no alternative but the evacuation of barracks and their conversion into hospitals. This had been planned in peace time, but it could not be carried out in a moment, especially during the hot weather. You must remember, too, that a very great percentage of our Indian soldiers were away on leave at the time of the outbreak, and their immediate recall threw an additonal burden on the railways, already strained to almost their limits.

The Commander-in-Chief has informed us that his experience in the Army has led him to the conclusion that a certain time must always elapse before the machinery to ensure smooth working of hospital and rearward services materialises, however elaborate be the pre-arranged schemes, and that the early days in France and Gallipoli will bear this out.

With regard to Kohat and the inadequacy of the hospital arrangements there, you can easily figure to yourself that when a station with hospital accommodation to meet the needs of a few battalions had to be expanded to requirements of a division owing to serious menace to our left flank by Nadir Khan's advance to Thal, there must necessarily be some delay and inconvenience, especially as the amenities of civilisation are not forthcoming in what is little more than a frontier outpost. As soon as Nadir Khan had been dealt with, the British battalions were diverted to stations near the frontier, where there were barracks for British troops. The Officer-in-Command assures me that everything that was possible was done.

Outbreaks of cholera are unavoidable throughout India, and it is little to be wondered at that the frontier has not been immune. We may consider ourselves fortunate that the outbreak has not been worse, for the best of discipline is not proof against the pangs of thirst, and a thirsty man, especially a follower, in an Indian July does not pause to consider source at which he quenches his thirst.

A BAD CHOLERA EPIDEMIC.

Special complaints were made about the cholera camp at Kohat, mention being made of the absence of equipment, furniture, sheets, and ice, and that the food and cooking were very bad.

The reply is that during the epidemic there 400 cases had to be dealt with in fifteen days. Cholera hospitals were improvised with all possible speed on June 7, and were working satisfactorily on June 12. It is denied that there was any deficiency in hospital furniture, sheets, or ice. It is admitted that the cooking was not satisfactory at the onset, but " fully efficient cooks were provided for the Indian hospitals by June 12, and for the British hospitals by June 20 " (the epidemic ended on June 20).

This reply did not satisfy the Secretary of State. He telegraphed again: " Specific allegations have been made that for a few days there were no arrangements made for feeding officers, no sheets or basins, that an officer's private basin was seized and all patients washed in it, that service of food was bad. I cannot reconcile this with Kohat's statement that there was no deficiency in hospital furniture and sheets."

The reply to this was not received in time for inclusion in the White Paper, nor was a reply yet to hand to the following telegram from Mr. Montagu on August 7:

" Question in Parliament alleges representations made to General Staff, Simla, in 1917 regarding necessity for pipe line for water to camp site at Ali Masjid, but nothing done until outbreak of cholera recently. Please let me know facts."

